# Correlation of humoral immune responses to different SARS-CoV-2 antigens with virus neutralizing antibodies and symptomatic severity in a German COVID-19 cohort

**DOI:** 10.1080/22221751.2021.1913973

**Published:** 2021-04-08

**Authors:** Alexandra Rockstroh, Johannes Wolf, Jasmin Fertey, Sven Kalbitz, Stefanie Schroth, Christoph Lübbert, Sebastian Ulbert, Stephan Borte

**Affiliations:** a Fraunhofer Institute for Cell Therapy and Immunology, Leipzig, Germany; b Department of Laboratory Medicine, Hospital St. Georg, Leipzig, Germany; c ImmunoDeficiencyCenter Leipzig (IDCL) at Hospital St. Georg Leipzig, Jeffrey Modell Diagnostic and Research Center for Primary Immunodeficiency Diseases, Leipzig, Germany; d Department of Infectious Diseases/Tropical Medicine, Nephrology and Rheumatology, Hospital St. Georg, Leipzig, Germany; e Interdisciplinary Center for Infectious Diseases, Leipzig University Hospital, Leipzig, Germany; f Division of Infectious Diseases and Tropical Medicine, Department of Medicine II, Leipzig University Hospital, Leipzig, Germany

**Keywords:** COVID-19, neutralizing antibodies, inactivated SARS-CoV-2, humoral immunity, RBD-ELISA, nucleocapsid-ELISA, S1-ELISA

## Abstract

Monitoring the humoral protective immune response and its durability after SARS-CoV-2 infections is essential for risk assessment of reinfections, the improvement of diagnostic methods and the evaluation of vaccine trials. We have analyzed neutralizing antibodies and IgG responses specific to different antigens, including the inactivated whole-virion of SARS-CoV-2, the spike subunit 1 protein and its receptor binding domain, as well as the nucleocapsid protein. We show the dynamic developments of the responses from the early convalescent stages up to 9 months post symptoms onset in follow-up samples from 57 COVID-19 patients with varying clinical severity. By correlating antibody signals to neutralizing titres, valid diagnostic markers for the estimation of neutralizing protection could be identified.

## Introduction

The novel severe acute respiratory syndrome coronavirus 2 (SARS-CoV-2) has emerged in December 2019 in Wuhan, China causing a severe acute respiratory disease (COVID-19) [1]. The virus has rapidly spread into a pandemic with over 100 million confirmed cases and over 2.6 million deaths worldwide (as of March 2021). Symptoms range from absent or mild “common cold” appearance to critical cases with severe pneumonia, associated with high mortality due to acute respiratory failure [2]. Natural antibodies to SARS-CoV-2 target structural and non-structural proteins such as the nucleocapsid (N) and spike (S) proteins, which are therefore utilized in serological tests [3]. N is a highly abundant viral protein, which is located inside the viral envelope and encloses the viral genome. In contrast, the S protein is a target of neutralizing antibodies of which the majority binds to the receptor-binding domain (RBD), located at the tip of trimeric S at the viral surface [4,5]. The S protein can be divided into different sub-units, the N-terminal S1 domain that includes RBD and C-terminal S2 domain which mediates cell membrane fusion and is known to be less specific in serological assays than S1 due to higher homology to other human coronaviruses [6]. Due to the very recent emergence of the disease, information on the long term-course of immunity to SARS-CoV-2 is very limited. However, understanding of the duration and dynamics of protective immune responses is essential for a proper risk evaluation of re-infections and the implementation of effective control measures. These include vaccines which are currently being licensed or are already in mass use [7]. Previous studies attending several months post symptom onset (PSO) have demonstrated the robustness of the humoral response following a SARS-CoV-2 infection [8,9]. Here, we studied neutralizing and antigen-specific antibody responses in a cohort of symptomatic COVID-19 patients in early convalescent and follow-up stages up to 9 months PSO and evaluate the applicability of different antigens as diagnostic correlates of neutralizing antibody protection.

## Material and methods

### Serum samples

A total of 57 patients admitted between March 3 and May 25, 2020 to the Department of Infectious Diseases/Tropical Medicine, Nephrology and Rheumatology or to the outpatient department at Hospital St. Georg in Leipzig, Germany and were followed-up up to 6–9 months PSO (Median 7.9 months, IQR 6.6–8.0). The first group consists of 38 non-hospitalized COVID-19 patients with mild outcome: uncomplicated upper airway symptoms without requirement of supplemental oxygen and none-respiratory symptoms. The second group includes 19 hospitalized patients with severe symptoms (*n* = 15): receiving supplemental oxygen and critical cases (*n* = 4): receiving ventilatory support, multiple organ failure. SARS-CoV-2 virus particles were detected by RT–PCR. The ethics committee of the Saxonian medical chamber approved the study (registry number EK-allg-37/10–1). Controls included samples (*n* = 100) from blood donors taken before 2020 (kindly provided by Jonas Schmidt-Chanasit, Bernhard Nocht Institute, Hamburg). Written informed consent was obtained from all study participants.

### SARS-CoV-2 RT–PCR

To detect SARS-CoV-2 virus particles, either nasopharyngeal swabs (Copan Liquid Amies eSwabs) or pharyngeal lavage specimens were analyzed by RT–PCR. Specimens were subjected to cellular lysis and RNA extraction on a MagNA Pure 24 System (Roche) or QiaSymphony (Qiagen). Real-time RT–PCR was conducted using LightCycler Multiplex RNA Virus Master Mix on a Lightcycler 480 RT system (both Roche) or a ViiA7 system (Applied Biosystems). For SARS-CoV-2 analysis, the Sarbecovirus specific LightMix Modular SARS-CoV (COVID-19) E gene assay was used (TIB Molbiol). EAV control (TIB Molbiol) was used as extraction and internal PCR control. All (RT)-PCR reactions were performed according to manufacturer's protocol.

### SARS-CoV-2 virus culture, purification and inactivation

All experiments containing the active SARS-CoV-2 were performed in the BSL-3 facilities of Fraunhofer Institute for Cell Therapy and Immunology, Leipzig. Vero E6 cells were grown in T175 flasks to a confluence of approx. 80–90% and were infected at a multiplicity of infection of 0.001 SARS-CoV-2 (isolate BetaCoV/Germany/BavPat1/2020, obtained from the European Virus Archive Global, EVAg) focus forming units per cell in 5 ml serum free Dulbecco's modified Eagle's medium (DMEM). After 1 h at 37°C, 20 ml of DMEM with 2% FCS was added and cells were incubated for two days at 37°C with 5% CO_2_ until cytopathic effect (CPE) was visible. Virus containing supernatant was first centrifuged at 4000 g for 10 min at 4°C and then purified by ultracentrifugation on a 30% sucrose cushion in MSE buffer (10 mM MOPS, pH 6.8, 150 mM NaCl, 1 mM EDTA) at 25,000 rpm for 3.5 h and 4°C. The pellet was resuspended in MSE buffer and centrifuged at 10,000 g for 5 min and 4°C. Purified SARS-CoV-2 viral particles were chemically inactivated with 0.1% beta-propiolactone at 22°C. After 16–18 h beta-propiolactone was hydrolysed at 37°C for 2 h. Inactivation was validated by inoculation of 50 µl of inactivated SARS-CoV-2 on confluent Vero E6 cell monolayers in 6-well plates. The cells were then cultured at 37°C for 4 days and supernatants were passaged 1:1 on fresh Vero E6 cells with an additional incubation of 4 days at 37°C.

### SARS-CoV-2 neutralization assay

Heat-inactivated human serum samples were serially diluted in DMEM without FCS from 1:10 to 1:5120 in duplicates and incubated with 50–100 focus forming units of SARS-CoV-2 for 1 h at 37°C before addition to confluent Vero E6 monolayers in 96-well plates. After an incubation of 1 h at 37°C, supernatant was removed, cells were washed with PBS, overlaid with 1.2% Methyl cellulose in DMEM with 2% FCS and incubated for 24–26 h at 37°C in 5% CO_2_. Cells were then fixed with 4% paraformaldehyde in PBS for 15–30 min at room temperature, permeabilized and blocked with Perm-Wash buffer (0.1% saponin, 0.1% BSA in PBS). SARS-CoV-2 focus forming units were stained using a monoclonal human anti-S1 antibody (CR3022, abcam, 1:2,000) and a secondary goat anti-human IgG HRP-conjugated antibody (Dianova, 1:1,000). After the addition of TrueBlue substrate (Seracare), spots were counted with an ELISpot reader (AID Diagnostika). FRNT_90_ titres were determinded for each replicate as the reciprocal of the last dilution providing a minimum of 90% neutralization of focus forming units in comparison to virus control without serum. Mean FRNT_90_ values were calculated from duplicates for each serum sample. A positivity cut-off of FRNT_90_ ≥20 was determined with negative reference sera (*N* = 47), which were collected before 2020, data not shown.

### Protein expression and purification

SARS-CoV-2 RBD (amino acid residues 329–538 of spike protein, strain Wuhan-Hu-1) was cloned into pMT/BiP/V5 vector (Invitrogen) and stably transfected into *Drosophila S2* cells. For an expression culture, cells were seeded at a cell density of 5 × 10^6^ cells/ml in 600 ml Sf900II medium in 2 l baffled Erlenmeyer shaker flasks at 28°C and 90 rpm and were induced with 700 µM CuSo_4_. After 7 days the suspension culture was centrifuged for 15 min and 4000 g at 4°C and culture supernatant was concentrated and diafiltrated against His-binding buffer (20 mM sodium phosphate, 500 mM NaCl, 10 mM Imidazole, pH7,4) using Vivaflow 50R TFF cassettes (Sartorius) according to manufacturers’ instructions. SARS-CoV-2 RBD was purified by immobilized metal affinity chromatography (IMAC) with 5 ml HisTrap FF crude columns (GeHealthcare) and size exclusion chromatography with a 16/600 HiLoad Superdex 200 pg column (GeHealthcare) using the ÄKTA pure 25 l chromatography system (GeHealthcare).

### ELISA

For the detection of IgG-antibodies to the whole-virion of SARS-CoV-2 and SARS-CoV-2 RBD, Nunc PolySorp plates were coated with 1.5 µl per well of inactivated SARS-CoV-2 viral particles and 250 ng/well of RBD protein in 100 µl per well of carbonate coating buffer (15 mM Na_2_CO_3_, 7 mM NaHCO_3_ pH 9.6) respectively overnight at 4°C. The plates were then washed with PBS-0.05% Tween and blocked with 5% skim milk powder in PBS for 2 h at room temperature. After another wash step, 1:100 diluted human sera were incubated for 1.5 h at room temperature. Following a third wash step, a HRP-conjugated secondary goat anti human IgG antibody (Dianova, 1:20,000) or goat anti human IgG + IgM + IgA H&L antibody (Abcam, 1:10,000) was added for 1 h at room temperature. TMB substrate (Biozol) was added after a final wash step and incubated for 25 minutes before the reaction was stopped with 1 M H_2_SO_4_. Absorbance was detected at 450 nm with 520 nm as reference in a microplate reader (Tecan). The cut-off values were determined for each antigen individually and were validated using 100 pre-pandemic serum samples (Supplementary Figure 1). The positivity cut-off for inactivated whole virus (IWV) IgG was determined as the mean plus 2 SD of a set of 10 negative reference sera on each test plate. For RBD IgG positivity the cut-off was determined as the mean plus 6 SD of a set of 8 negative reference sera on each plate. For RBD IgGAM positivity the cut-off was determined as the mean plus 5 SD of 4 negative reference sera. Signal to cut-off ratios were then displayed and ratios higher 1 were considered as positive (Supplementary Figure 1). All measurements were performed at least in duplicates.

S1- and nucleocapsid specific IgG antibodies were detected with commercially available SARS-CoV-2-IgG CE-IVD labelled ELISA kits from Euroimmun and Virotech respectively. Measurements were performed on an automated ELISA processor (DSX, Dynex Technologies, UK).

### Statistical analysis

GraphPad Prism 6 (GraphPad Software Inc.) was used to analyze spearmen correlations between SARS-CoV-2 neutralization and different ELISA tests. Statistical tests were calculated as paired or unpaired two-way ANOVA. Categorical variables were given as frequencies or percentages with 95% Wilson-confidence intervals (CI_95%_). Fisher's exact test was applied for comparison of categorical variables.

## Results

We analyzed neutralizing and IgG antibody responses to different SARS-CoV-2 antigens of 57 individuals with PCR-confirmed SARS-CoV-2 infections with either mild or severe COVID-19 outcomes. The samples derived from an early-convalescent stage ≤2 months PSO (median 37 days PSO, IQR 31–46) and a follow-up 6–9 months PSO (median 238 days PSO, IQR 200–262) (Table 1).

**Table 1.** Study participants characteristics.

	*N*	Age		Gender		Timepoint of blood collection			
		Median (Min-Max)	IQR	Male (%)	Female (%)	≤2 months PSO		6–9 months PSO	
						Median (Min-Max)	IQR	Median (Min-Max)	IQR
Total	57	51 (7–82)	43–61	49.1	50.9	37 (7–65)	31–46	238 (173–304)	200–264
Mild	38	44 (7–80)	39–61	55.3	44.7	37 (7–65)	33–46	239 (173–304)	195–262
Severe	19	64.53 (46–82)	59–77	36.8	63.2	35 (8–60)	17–49	221 (176–298)	202–267

Abbreviations: IQR: Interquartile range; PSO: post symptom onset.

**Figure 1.**
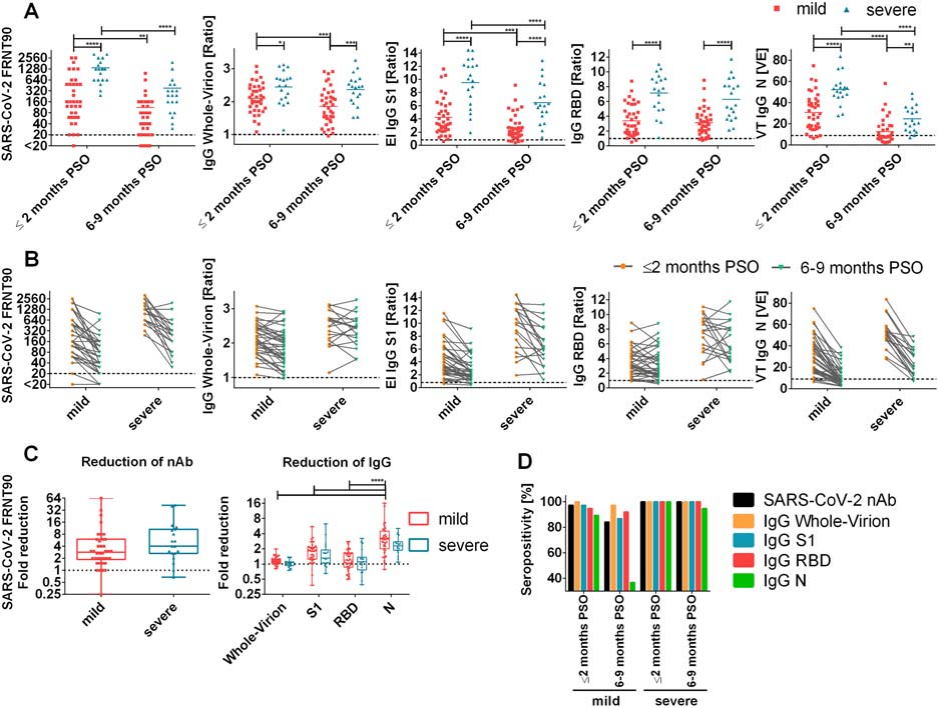
Antibody characteristics of patient sera with mild and severe COVID-19 outcomes as measured on different time points PSO; A: SARS-CoV-2 focus-reduction-neutralizing titres (FRNT90) and IgG-specific antibodies to SARS-CoV-2 whole-virion, S1, RBD and N protein clustered ≤2 months PSO and 6–9 months PSO; two-way ANOVA with Holm-Sidak's multiple comparison test * = *p* < 0.05, ** = *p* < 0.01, *** = *p* < 0.001, **** = *p* < 0.0001, B: Individual progression of SARS-CoV-2 FRNT90 and IgG-specific antibodies to SARS-CoV-2 whole-virion, S1, RBD and N protein of mild and severe COVID-19 patients; C: Reduction of SARS-CoV-2 neutralizing titres and IgG antibody signals specific to SARS-CoV-2 whole-virion, S1, RBD and N protein over two follow-up timepoints, calculated as ratio of value on timepoint 1 (≤2 months PSO) to timepoint 2 (6–9 months PSO); D: Seropositivity (%) of study participants for SARS-CoV-2 neutralizing antibodies and IgG on SARS-CoV-2 whole-virion, S1, RBD and N; Abbreviations: ANOVA: 1-way analysis of variance; COVID-19: coronavirus induced disease 19; EI: Euroimmun; FRNT90: Focus reduction neutralization titre 90; N: nucleocapsid protein; nAb: neutralizing antibodies; PSO: post symptom onset; RBD: receptor binding domain; SARS-CoV-2: Severe acute respiratory syndrome coronavirus-2; S1: subunit 1 of SARS-CoV-2 spike protein; VT: Virotech.

**Figure 2.**
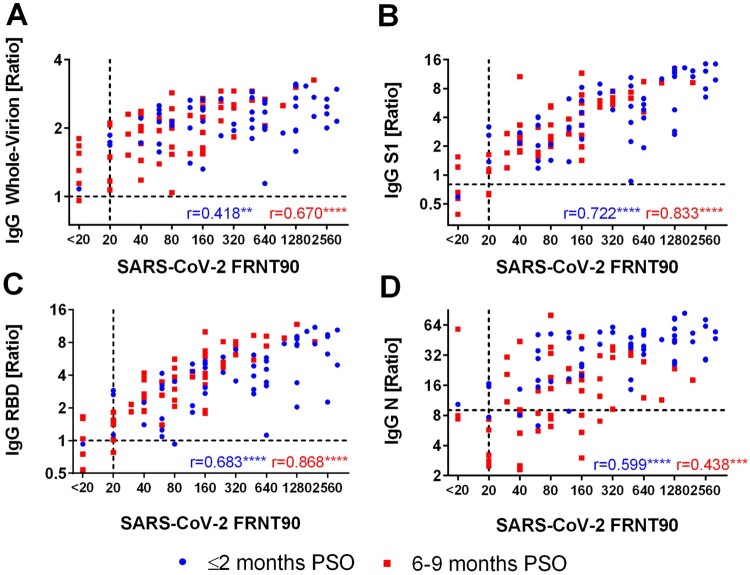
Correlation of SARS-CoV-2 neutralization titres (FRNT90) to A: SARS-CoV-2 whole-virion IgG ELISA; B: S1 IgG ELISA; C: RBD IgG ELISA and D: N IgG ELISA for samples ≤2 months PSO and 6–9 months PSO; Abbreviations: FRNT90: Focus reduction neutralization titre 90; N: nucleocapsid protein; PSO: post symptom onset; r: Spearman's rank correlation coefficient; RBD: receptor binding domain; SARS-CoV-2: Severe acute respiratory syndrome coronavirus-2; S1: subunit 1 of SARS-CoV-2 spike protein; * = *p* < 0.05, ** = *p* < 0.01, *** = *p* < 0.001, **** = *p* < 0.0001.

**Figure 3.**
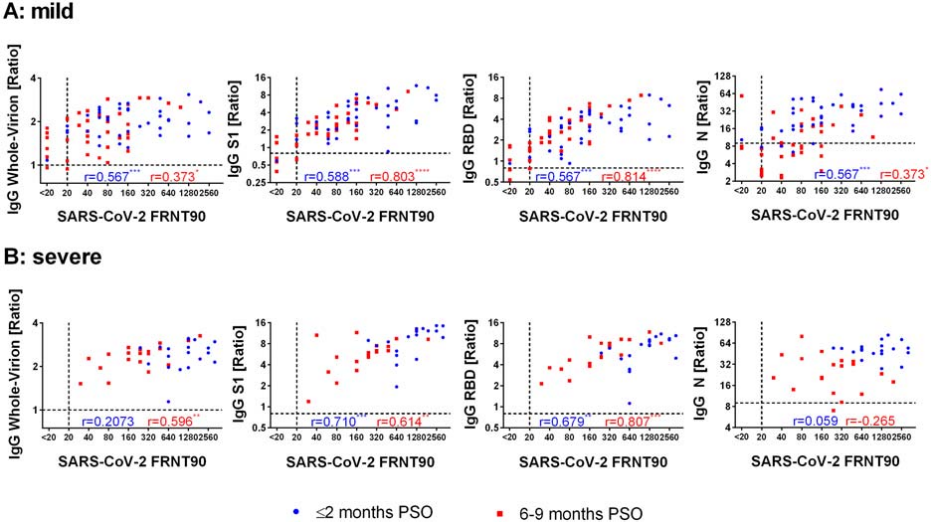
Correlation of SARS-CoV-2 neutralization titres (FRNT90) of A: mild COVID-19 patients and B: severe COVID-19 patients to SARS-CoV-2 whole-virion IgG ELISA, S1 IgG ELISA, RBD IgG ELISA and N IgG ELISA Abbreviations: COVID-19: corona virus induced disease 19; FRNT90: Focus reduction neutralization titre 90; N: nucleocapsid protein; PSO: post symptom onset; r: Spearman's rank correlation coefficient; RBD: receptor binding domain; SARS-CoV-2: Severe acute respiratory syndrome coronavirus-2; S1: subunit 1 of SARS-CoV-2 spike protein; * = *p* < 0.05, ** = *p* < 0.01, *** = *p* < 0.001, **** = *p* < 0.0001.

**Figure 4.**
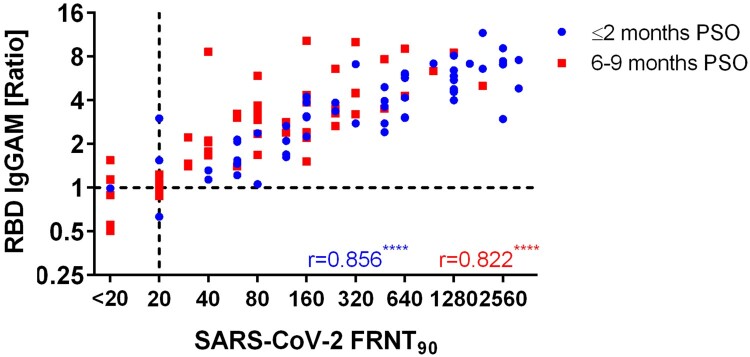
Correlation of SARS-CoV-2 neutralization titres (FRNT90) to RBD IgGAM ELISA for samples ≤2 months PSO and 6–9 months PSO; Abbreviations: FRNT90: Focus reduction neutralization titre 90; PSO: post symptom onset; r: Spearman's rank correlation coefficient; RBD: receptor binding domain; SARS-CoV-2: Severe acute respiratory syndrome coronavirus-2; **** = *p* < 0.0001.

Neutralizing antibody titres in early-convalescent sera strongly correlated with disease severity of the patients with mean titres of 449 (148%CV) and 1368 (67%CV) for mild and severe outcomes, respectively (Figure 1A). Follow-up samples from the severe group presented a significant decrease in neutralizing titres (Median 4-fold reduction, IQR 2.7–10.7), whereas titres of patients showing mild symptoms only slightly decreased (Median 2.8-fold reduction, IQR 1.9–6). In the late-convalescent stage, mean titre differences between the groups were statistically insignificant (Figure 1A-D). However, 13.16% (5/38) of the mild group sero-reverted for SARS-CoV-2 neutralization, whereas all severe patients remained positive for neutralizing antibodies at 6–9 months PSO (Figure 1D). One patient did not develop neutralizing antibodies at all. Next, we analyzed the binding of IgG to different antigens, namely inactivated whole virus (IWV), recombinant S1, RBD and N proteins. Similar to the neutralizing response, we observed a significant positive correlation of the disease severity on mean IgG-levels to all of these antigens (Figure 1A). IgG binding to IWV showed the least difference between the mild and the severe group and only slightly decreased in mild patients in the follow-up course. In addition, IWV was significantly more sensitive and presented a significantly lower mean signal reduction over time compared to N protein. However, IWV IgG ELISA showed 15% cross-reactions with pre-pandemic sera. (Figure 1C-D, Supplementary Table 2). IWV, S1-, RBD- and N- specific IgG antibody signals showed a comparable increase in dependence of disease severity. Of note, only S1- and N- specific antibodies waned 6–9 months PSO whereas RBD-specific IgG antibodies remained stable or even increased in the follow-up samples (Figure 1A-C). In consequence, due to the excessive reduction of nucleocapsid IgG antibodies, this assay showed lowest sensitivity in the mild group 6–9 months PSO with only 36.8% (14/38) sero-positivity (Figure 1D). Subsequently, the correlation of SARS-CoV-2 neutralizing antibody titres with different antigen-specific IgG responses was evaluated (Figure 2A-D) and we found a best positive correlation with S1- and RBD- reactive IgG antibodies. Furthermore, due to the high correlation of S1- and RBD- specific IgG antibodies to SARS-CoV-2 neutralizing titres, estimation of specific ELISA thresholds which still represent neutralizing antibodies with a probability of ≥99% (95%CI 93.6–99.9) was possible (Supplementary Table 1). However, the correlation was higher in samples 6–9 months PSO, particularly due to low or negative S1- and RBD-IgG signals in some samples from the early group, despite of high neutralizing antibody titres. For these samples, the correlation was slightly enhanced for the S1 antigen. In contrast, for patients who had severe symptoms RBD- specific IgG antibodies correlated better with neutralizing antibodies 6–9 months PSO than S1-IgG antibodies because some samples showed high S1-IgG responses despite of low neutralizing antibody titres (Figure 3). Similar to RBD- and S1-, SARS-CoV-2 whole-virion specific IgG antibodies displayed a better correlation to neutralization 6–9 months PSO than in the early phase of convalescence (Figure 2B). In contrast, antibodies specific to N protein indicated only a weak correlation to neutralization 6–9 months PSO (Figure 2D), especially because of the fast decrease of such antibodies in late convalescent samples. Nevertheless, in samples taken ≤2 months PSO, nucleocapsid- IgG antibodies showed a better correlation with neutralizing antibodies than whole-virion IgG. To analyze more exactly the correlation of ELISA signals to neutralizing titres in early convalescent samples, IgA and IgM antibody detection was included in the RBD ELISA in addition to IgG (RBD IgGAM). In this setup signals from samples with low RBD IgG signals and high neutralizing titres were enhanced and correlated with FRNT90 fold reduction over time (Figure 4, Supplementary Figure 2).

## Discussion

We have analyzed the temporal dynamics of humoral immune responses to SARS-CoV-2 in a cohort of German patients with either mild or severe disease symptoms for up to 9 months after symptom onset. Our results indicate that neutralizing antibody titres, as well as antibodies binding to whole-virion, S1-, RBD- and nucleocapsid proteins are highest in early convalescent sera of COVID-19 patients with severe symptoms, as compared to patients with mild symptoms. This observation is in line with previous studies [10,11] and might result from higher viral loads or prolonged viral shedding in severely affected patients, leading to extended antigen presentation and presumably stronger antibody responses [12]. During 6–9 months after symptom onset, mean neutralizing antibody titres decreased and were still pronounced in the severe group, although the difference to the group with mild symptoms was statistically insignificant. However, neutralizing antibodies waned in 13% of patients with mild symptoms below the detection limit, whereas in all severely affected individuals neutralizing antibodies remained detectable. Yet, it has to be clarified whether this would have consequences for potential re-infections or if memory B- and T-cells could provide sustainable protection against COVID-19 [9,13]. Despite lower mean neutralizing titres when compared to severe outcomes in early convalescence, patients with mild symptoms presented a relatively broad titre range and a relatively slow decay of neutralizing antibodies over time. Here, several studies are discordant, as they generally show low responses in asymptomatic and mild COVID-19 patients [10,14–16]. A possible explanation could be the missing standardization between laboratories. Regarding neutralizing antibodies, these are often evaluated using pseudotyped viruses or RBD-based assays. However, this may not reflect the original virus structure and/or measure only one portion of neutralizing antibodies in contrast to neutralization assays with active virus [17,18], as it has been used in this study. Recently, it has been shown that neutralizing antibody responses are very individual and diverse, especially in mild to moderate symptomatic patients. Restriction to the receptor-binding domain of the spike protein is doubtful and many patients might feature other neutralizing responses, such as an interplay between S1- and S2-specific antibodies [10,19,20]. This corresponds with the findings shown here, as S1- and RBD- IgG signals correlated best with SARS-CoV-2 neutralization. However, this correlation is weakest in the early convalescent stage, which indicates different dynamics for these antibody subsets in the early protection against SARS-CoV-2 and an improvement of the neutralizing capacity of RBD IgG antibodies over time. We have shown that the addition of IgM and IgA antibody detection leads to an improvement of the correlation to neutralizing titres, indicating the contribution of these antibody classes to neutralization, which is supported by other studies [21–24]. We have analyzed different antigens and found the maximal sensitivity with IgG antibodies against the inactivated whole-virion of SARS-CoV-2. These signals contain antibodies directed to many structural and quaternary epitopes on the outer virus surface, including M- and E- proteins and the trimeric form of spike [25]. This is in line with other studies reporting that serological assays based on stabilized trimeric S proteins lead to highest sensitivities [26]. Nevertheless, specificities of negative pre-pandemic controls needs to be evaluated critically for the IWV ELISA (Supplementary Figure 1), because it suggests cross-reactivity from antibodies to other human coronaviruses, as was also reported for the trimeric S [27]. Furthermore, we show a rapid decay of nucleocapsid specific IgG antibodies 6–9 months PSO in patients with mild symptoms, which underlines the less favourable single-use of this antigen as a diagnostic tool for epidemiological follow-up studies [28,29].

In summary, our investigations prove the durability of neutralizing and virus-specific antibodies at least 9 months PSO following a SARS-CoV-2 infection. We demonstrate that indirect S1- and RBD- IgG/IgGAM ELISAs represent valid diagnostic markers to estimate levels of neutralizing antibodies up to 9 months PSO. This might have implications for the assessment of epidemiological control measures and the future evaluation of protective immunity after vaccination.

## Supplementary Material

Clean_copy_of_supplementary_material.docx
