# Supplementary material for: Correlation of humoral immune responses to different SARS-CoV-2 antigens with virus neutralizing antibodies and symptomatic severity in a German COVID-19 cohort
Source: Emerg Microbes Infect. 2021 Apr 8;10(1):774–81. doi: 10.1080/22221751.2021.1913973 (PMC8079054; doi:10.1080/22221751.2021.1913973)
Supplement: Clean_copy_of_supplementary_material.docx [file TEMI_A_1913973_SM4597.docx]

# Supplementary material

# Supplementary Tables

Supplementary Table 1: Specific cut-off values for S1- and RBD- IgG ELISAs for the detection of neutralizing antibodies after Covid-19 diagnosis using a SARS CoV-2 virus neutralization assay as reference

|  | S1-IgG  [Ratio] | RBD-IgG  [Ratio] |
| --- | --- | --- |
| **Threshold** | >1.224 | >1.65 |
| **True positive** | 101 | 96 |
| **False positive** | 1 | 1 |
| **True negative** | 6 | 6 |
| **False negative** | 7 | 12 |
| **PPV (CI_95%_)** | 99.0  (93.9-99.9) | 99.0  (93.6-99.9) |

PPV: positive predictive value, S1: subunit 1 of SARS-CoV-2 spike protein, RBD: receptor binding domain

Supplementary Table 2: Test sensitivities of IgG IWV, IgG S1, IgG RBD, IgG N, IgGAM RBD and SARS-CoV-2 FRNT90 for different timepoints of sample collection and COVID-19 severities

|  |  |  | ***IgG IWV*** | | | ***IgG S1*** | | | ***IgG RBD*** | | | ***IgG N*** | | | ***IgGAM RBD*** | | | ***SARS-CoV-2 FRNT90*** | | |
| --- | --- | --- | --- | --- | --- | --- | --- | --- | --- | --- | --- | --- | --- | --- | --- | --- | --- | --- | --- | --- |
| ***Timepoint*** | ***Severity*** | ***Total*** | ***N*** | ***%*** | ***95% CI*** | ***N*** | ***%*** | ***95% CI*** | ***N*** | ***%*** | ***95% CI*** | ***N*** | ***%*** | ***95% CI*** | ***N*** | ***%*** | ***95% CI*** | ***N*** | ***%*** | ***95% CI*** |
| ***≤2 months PSO*** | *Total* | 57 | 57 | 100 | 93.73 - 100 | 56 | 98.24 | 90.61 - 99.96 | 55 | 96.49 | 87.89 - 99.57 | 53 | 92.98 | 83.00 - 98.05 | 55 | 96.49 | 87.89 - 99.57 | 56 | 98.24 | 90.61 - 99.96 |
|  | *Mild* | 38 | 38 | 100 | 90.75 - 100 | 37 | 97.36 | 86.19 - 99.93 | 36 | 94.73 | 82.25 - 99.36 | 34 | 89.47 | 75.20 - 97.06 | 36 | 94.73 | 82.25 - 99.36 | 37 | 97.36 | 86.19 - 99.93 |
|  | *Severe* | 19 | 19 | 100 | 82.35 - 100 | 19 | 100 | 82.35 -100 | 19 | 100 | 82.35 -100 | 19 | 100 | 82.35 -100 | 19 | 100 | 82.35 -100 | 19 | 100 | 82.35 -100 |
| ***6-9 months PSO*** | *Total* | 57 | 56 | 98.24 | 90.61 - 99.96 | 52 | 91.22 | 80.70 - 97.09 | 54 | 94.73 | 85.38 - 98.90 | 32 | 56.14 | 68.75 - 93.98 | 52 | 91.22 | 80.70 - 97.09 | 51 | 89.47 | 78.48 - 96.04 |
|  | *Mild* | 38 | 37 | 97.36 | 86.19 - 99.93 | 33 | 86.84 | 71.91 - 95.59 | 35 | 92.10 | 78.62 - 98.34 | 14 | 36.84 | 42.36 - 69.26 | 33 | 86.84 | 71.91 - 95.59 | 32 | 84.21 | 68.75 - 93.98 |
|  | *Severe* | 19 | 19 | 100 | 82.35 -100 | 19 | 100 | 82.35 -100 | 19 | 100 | 82.35 -100 | 18 | 94.73 | 73.97 - 99.87 | 19 | 100 | 82.35 -100 | 19 | 100 | 82.35 -100 |
| ***Total*** | *Total* | 114 | 113 | 99.12 | 95.21 - 99.98 | 108 | 94.73 | 88.90 - 98.04 | 109 | 95.61 | 90.06 - 98.56 | 85 | 74.56**** | 65.55 - 82.25 | 107 | 93.85 | 87.76 - 97.50 | 107 | 93.85 | 87.76 - 97.50 |

Fisher’s Exact Test: ****=p<0.0001

Abbreviations: FRNT90: Focus reduction neutralization titer 90; IWV: Inactivated Whole-Virion; N: SARS-CoV-2 nucleocapsid protein; PSO: post symptom onset; RBD: receptor binding domain; SARS-CoV-2: Severe acute respiratory syndrome coronavirus-2; S1: subunit 1 of SARS-CoV-2 spike protein; 95% CI: 95 % confidence interval

Supplementary Fig. 1: Reactivity of antibodies of different human serum samples on A: SARS-CoV-2 inactivated whole-virion IgG B: SARS-CoV-2 RBD IgG and C: RBD IgGAM; Abbreviations RBD: receptor binding domain; SARS-CoV-2: Severe acute respiratory syndrome coronavirus-2;

Supplementary Fig.2: Correlation between fold reduction of SARS-CoV-2 FRNT90 and A: S1 IgG, B: RBD IgG and C: RBD IgGAM
Abbreviations FRNT90: Focus reduction neutralization titer 90; r: Pearson r; RBD: receptor binding domain; S1: subunit 1 of SARS-CoV-2 spike protein; SARS-CoV-2: Severe acute respiratory syndrome coronavirus-2;* = p < 0.05, ** = p < 0.01, **** = p < 0.0001;
